# Imaging hypoxia in endometrial cancer: How and why should it be done?

**DOI:** 10.3389/fonc.2022.1020907

**Published:** 2022-11-09

**Authors:** Nandita M. deSouza, Ananya Choudhury, Mel Greaves, James P. B. O’Connor, Peter J. Hoskin

**Affiliations:** ^1^ Division of Radiotherapy and Imaging, The Institute of Cancer Research, London, United Kingdom; ^2^ Department of Imaging, The Royal Marsden National Health Service (NHS) Foundation Trust, London, United Kingdom; ^3^ Radiation Oncology, The Christie National Health Service (NHS) Foundation Trust Manchester, Manchester, United Kingdom; ^4^ The Division of Cancer Sciences, University of Manchester, Manchester, United Kingdom; ^5^ Centre for Evolution and Cancer, The Institute of Cancer Research, London, United Kingdom; ^6^ Radiation Oncology, Mount Vernon Cancer Centre, Northwood, United Kingdom

**Keywords:** hypoxia, endometrial cancer, magnetic resonance imaging (MRI), oxygen-enhanced MRI, molecular subtypes

## Introduction

Tissues become hypoxic when their oxygen consumption exceeds their supply. In tumors, neoangiogenesis results in disordered vascular morphology and this leads to inadequate oxygen supply to rapidly growing cells (1). However, because vessels have variations in their vascular tone, hypoxic status is a dynamic rather than a static event; it may be transient and is often cyclical (2). Cyclical hypoxia with a median periodicity of 15 mins has been described in xenografts and head and neck cancers in patients (3). Hypoxia is important because it is well accepted as a poor prognostic factor in for patients with a range of cancer types (4–7) where it has been associated with disease that is progressive, resistant and metastatic (8).

Under hypoxic conditions, oxygen-sensitive transcription factors (hypoxia inducible factors [HIFs]) are upregulated. In the endometrium, HIF1α expression increases as the tissue undergoes changes from normality to being premalignant and then to become adenocarcinoma. This is paralleled by increased angiogenesis in the endometrium, suggesting that HIF1α and thus tissue hypoxia might be a key regulator in endometrial carcinogenesis (9). A poorer prognosis in patients with EC expressing HIF-1α has been demonstrated in a metaanalysis with a hazard ratio of 2.29 (10), and its link to tumor aggressiveness at other cancer sites is documented (11). Hypoxic status also has been associated with mutations of multiple genes in endometrial cancer (EC) (12). In other cancer types, driver mutations in p53, MYC and PTEN are enriched in hypoxic tumors (13) with an effect of hypoxia on mutational load (14). Therefore, as hypoxia is likely to determine the evolutionary trajectories and as a result the management and outcomes of cancer, imaging tumor hypoxic status in EC may offer prognostic value and facilitate personalisation of treatment strategies (15).

## Endometrial cancer: Diagnosis, staging and the changing molecular landscape

EC, (9,300 new cases per annum in the UK (16), 65,620 new cases in 2020 in USA (17) usually presents with post-menopausal bleeding and is detected at an early stage. Diagnostic confirmation on pipelle sampling of the endometrium or at hysteroscopy is followed by pelvic magnetic resonance imaging (MRI) for disease staging (18). Endometrioid and mucinous carcinomas are classified as type I and serous and clear cell carcinomas as type II. The former are usually low grade and low stage at presentation and the latter high grade and advanced stage. Disease outcome depends on tumor grade, stage, subtype, depth of myometrial invasion, lymphovascular space invasion and lymph node involvement (19). In fact, in type I endometrial adenocarcinoma, high expression of HIF-1 α showed a significant correlation with higher grade of the tumor, depth of myometrial invasion, adnexal invasion and clinical stage (20), which strengthens the argument for hypoxia driving tumor progression by favouring selection of adverse genetic clones.

Molecular classification is now used to define risk groups in EC, namely deoxyribose nucleic acid (DNA) polymerase ɛ ultramutated (POLEmut), mismatch repair-deficient (MMRd), p53 mutant (p53abn) and those EC lacking any of these alterations, referred to as NSMP (non-specific molecular profile) (21). Prognosis is extremely good in POLE and poor in p53 mutant cancers (22) with poor clinical outcomes in the latter group being independent of histology grade or stage (23–25); the other two categories fall between these two extremes. TP53 mutations are highly prevalent in the serous (Type II) subtype (88% of 42 serous ECs (26), and are also present in a subset of endometroid (Type I) carcinomas (15% of 186 endometroid ECs (26). Recent data show that a subset of p53mut EC is homologous recombination‐deficient (HRD), and some of these EC can arise in the context of germline breast cancer (*BRCA)1/2* mutations (27–29). The exact prevalence of HRD in p53mut EC is currently unknown; in a small and selected set of cases it was 46% (28).

The biological and genetic mechanisms that causally link hypoxia with progression of disease are being unravelled. Hypoxia and associated acidosis activate the TP53 dependent stress response and apoptosis (30). This then provides selective evolutionary pressure for the emergence of mutants in the TP53 response (13). Such genetic variants then preferentially expand as a population. Their fitness benefit is however more than simple survival. They are intrinsically more resistant to many of the therapeutic modalities that operate *via* the TP53 apoptosis pathway. Their failure to undergo TP53 driven cell cycle arrest and DNA repair also leads to genetic instability (31). Additionally, as hypoxia mimics the mesenchymal stem cell niche (32), surviving TP53 mutants undergo Epithelial-Mesenchymal transition (EMT) to migratory, stem cell phenotypes. Hence the hypoxic microenvironment encourages the selection of cancer cell populations that have an expanded pool of stem cells (the critical units of selection in cancer progression) that are likely to be genetically unstable. These biological features fuel both disease progression and the likelihood of treatment resistance (33).

## Methodology and challenges of imaging hypoxia

The prognostic relevance of hypoxia in EC has been determined largely by using the expression of HIF-1α (12) and the presence of tumor necrosis (34). Although the correlation of HIF-1α with imaging estimates of hypoxia is variable (35), an association has been demonstrated in EC (15). On imaging, hypoxia may be measured indirectly or directly. Traditionally, tumor vasculature has been imaged using ultrasound and computerized tomography (CT). Doppler ultrasound, based on the frequency shift of moving echo-generating components in flowing blood, has been used to classify endometrial pathologies (36). With contrast-enhanced CT, extracted metrics relate to blood flow, blood volume and vascular permeability (37). Although increased vascularity in tumors is highly disorganised and leaky, often indicating an increased hypoxic status (38), it is not a direct measurement.

Positron Emission Tomography (PET) uses hypoxia-specific tracers such as ^18^F-labelled nitroimidazoles and copper (Cu)-labelled diacetyl-bis(N4-methylthiosemicarbazone) analogues (39). Under hypoxic conditions, free nitro radicals are retained within the cell. Though commonly used ^18^F-fluoroimidazole (^18^F-FMISO) (40) has relatively low uptake, slow kinetics and is influenced by non-hypoxic metabolism. ^18^F-FAZA [1-(5-fluoro-5-deoxy-α-D-arabinofuranosyl)-2-nitroimidazole)] offers better resolution and signal-to-noise ratio (41). Cu complex agents with diacetyl-bis(N4-methylthiosemicarbazone) (ATSM) ligand under hypoxic conditions cannot be reversibly oxidised by the cell also making Cu-ATSM a possible means for evaluating hypoxia in the clinic (42–44). However, its specificity is debatable and validation with pimonidazole stained tissues has been variable and tumor type specific (45, 46)

A shift to non-invasive hypoxia imaging with MRI is advantageous (47). In blood oxygen level dependent (BOLD) MRI, also known as intrinsic susceptibility-weighted MRI, paramagnetic deoxyhaemoglobin within red blood cells (in contrast to non-paramagnetic oxyhaemoglobin) increases the MR transverse relaxation rate (R2*, the inverse of the transverse relaxation time T2*), of water in blood and surrounding tissues. Variations in perfusion mean that the relationship between R2* and tissue pO_2_ is non-linear and perfusion dependent. Nevertheless, BOLD-MRI is sensitive to changes in pO_2_ within vessels and in tissues adjacent to perfused vessels (48, 49). R2* has been shown to correlate positively with tissue hypoxia score (HP5) and oxygen pressure (50) and with HIF-2α expression in colorectal cancer with different tumor stages (51, 52). Advantages of the BOLD-MRI technique for measurement of hypoxia are lack of need for externally administered contrast media, easy repeatability, near real-time visualisation of time-dependent changes and a measure independent of blood flow. Nevertheless, the variability of the measurement (53), means that measuring a change in R2* following an oxygen challenge may be preferable particularly as they have been shown to correlate strongly with pimonidazole staining in tumor models (54).

Oxygen in solution and deoxy Hb also affect the longitudinal relaxation rate of tissues (55) and are exploited in the technique of oxygen-enhanced (OE)- MRI, also known as tumor oxygen level dependent contrast (TOLD). Their effect on the rate of longitudinal proton relaxation (R1) can be enhanced by the inhalation of 100% O2 which results in an increase in the relaxation rate in normoxic tissues, primarily due to an increase in dissolved oxygen (56). A measurable signal change of up to 20% is achievable in normoxic tissues with 100% O_2_ inhalation on clinical scanners (57) that can distinguish them from hypoxic tissue (58). OE-MRI has been validated in pre-clinical studies (59, 60) and had initial clinical translation (61, 62) with promising results. MRI measures of hypoxia can be implemented as an extension of the imaging staging examination but require standardisation of image acquisition and analysis methodology prior to clinical use.

## Endometrial cancer– opportunities for adjusting management strategies to hypoxic status

Management of EC is primarily surgical as patients with uterus-confined low-risk disease are often cured by surgery. Prognostic factors that describe groups by their risk of recurrence (histological type and grade, age, tumor size, and lymphovascular space involvement (63) are used to determine need for adjuvant therapies. Several trials have compared external beam radiotherapy (EBRT) after surgery versus observation after surgery in intermediate and high-risk disease: the PORTEC 1 (64), ASTEC/EN5 (65) and Gynecologic Oncology Group (GOG) (66) trials all showed a reduced risk of vaginal and pelvic relapse though overall survival did not differ. PORTEC 2 then showed that equivalent locoregional control could be achieved with vaginal brachytherapy without the toxicity of EBRT, so that adjuvant brachytherapy is the standard-of-care in patients with intermediate-risk disease following surgery (67). More recently, the PORTEC-3 trial, concluded that molecular classification has strong prognostic value in high-risk EC, with significantly improved recurrence-free survival with adjuvant chemoradiotherapy compared to radiotherapy alone for p53abnormal tumors, regardless of histologic type (68).

Hypoxia imaging and its link to TP53 status offers potential to refine management strategies by selecting patients through prognostic stratification. Pre-operative hypoxia imaging could identify the women who would most benefit from adjuvant radiotherapy and select women who might benefit from EBRT rather than adjuvant brachytherapy alone. Also, in hypoxic tumors post-surgery, where there is residual disease, it may be possible to dose-escalate with either brachytherapy, external beam radiotherapy or the use of a radiosensitiser, or omit or dose de-escalation when tumor hypoxia is not demonstrated. In locally advanced endometrial cancer (stage III) treated primarily with chemoradiotherapy, hypoxia imaging may also indicate those who would benefit from hypoxia modification with a radiosensitiser. Drugs like carbogen and nicotinamide can be combined with radiation without increasing late toxicity but may improve survival outcomes as in muscle-invasive bladder cancer (69). The effect of hypoxia on the immune tumor microenvironment is complex, but it is likely to promote resistance to immune modulatory approaches (70).

Neoadjuvant chemotherapy (NAC) has primarily been trialled in patients with Stage 4 or metastatic disease at presentation with uterine papillary serous carcinomas (71) to facilitate optimal surgical cytoreduction. More recently this has been extended to endometrioid adenocarcinoma (72, 73) where the use of NAC to enable cytoreductive surgery resulted in an increased progression-free and overall survival (74). It may be possible to refine the use of NAC further if tumor hypoxic status along with tumor stage and volume were considered to select patients likely to respond. Finally, the association of hypoxia with genetic instability and DNA damage repair efficacy (supported by the prevalence of HRD in p53 mutated EC (28)), indicates that hypoxia imaging could be an important predictive selector for women who benefit from agents such as poly adenosine diphosphate ribose polymerase (PARP) inhibitors although their use in EC remains to be established.

As with EC in the primary setting, locally recurrent EC that shows a high degree of hypoxia may benefit from dose-escalation or use of a radiosensitiser. Development of prognostic models based on hypoxic status and molecular profiling (75) may change approaches to maintenance therapies and surveillance. Poor prognostic tumors at risk of progressive disease thus identified may benefit from a more aggressive surveillance strategy adapted to their risk. It may also enable implementation of future maintenance therapy approaches in suitable patient cohorts.

## Discussion and concluding remarks

**Figure 1:**
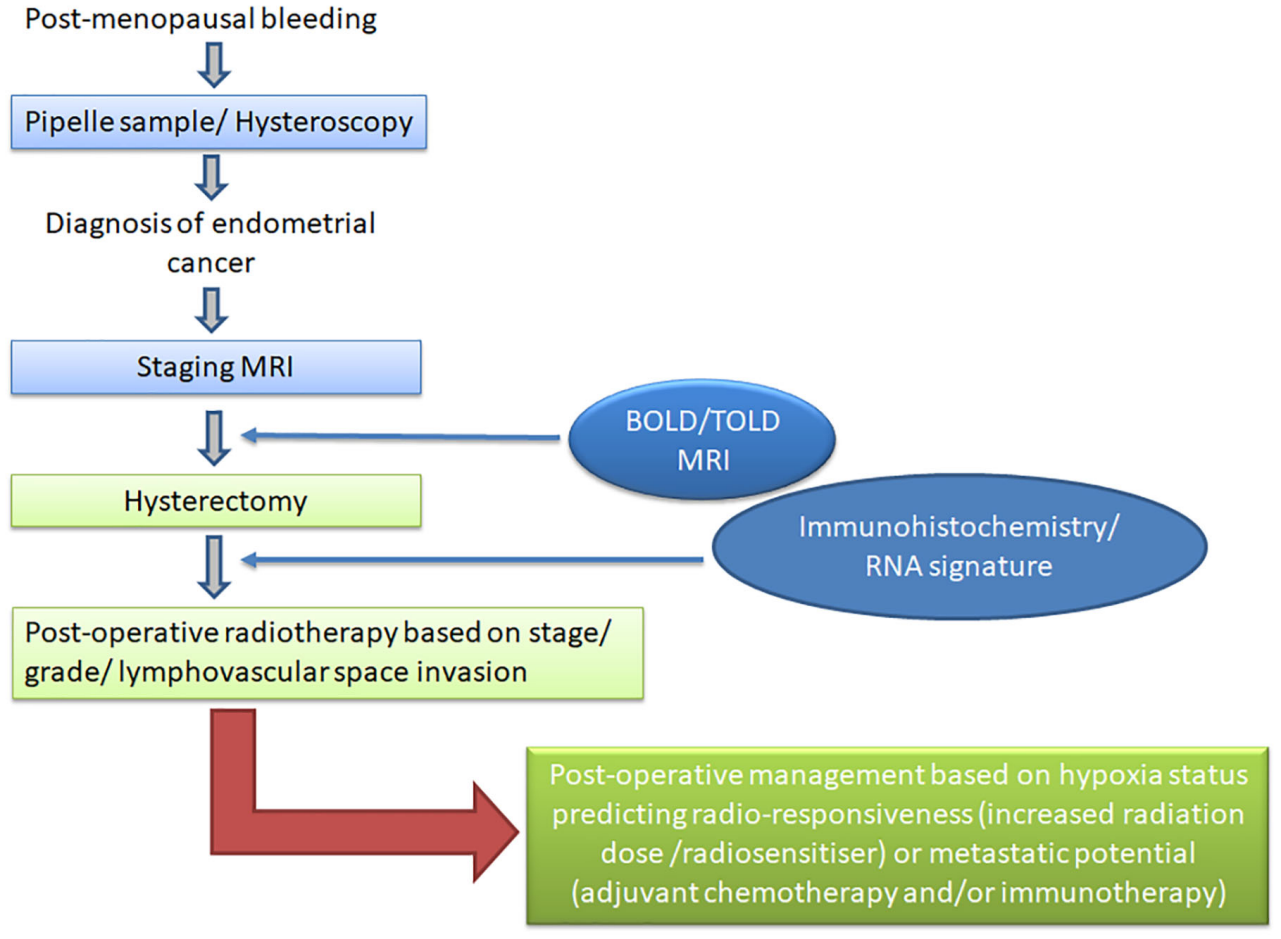
Diagnostic and management pathway for patients with endometrial cancer. Current diagnostic tests are shown in light blue and proposed hypoxia imaging in dark blue; current management options are shown in light green and alternative strategies based on hypoxia imaging and molecular stratification in dark green.

The technical validation and demonstration of target monitoring with hypoxia imaging remains a major challenge. Continuous measurements indicate variable O_2_ saturation, there is heterogeneity within each tumor and between tumor sites in the same patient (76) and thresholds for differentiating normoxic from hypoxic tissues in imaging studies are lacking. The impact of hypoxia on chemoresistance has been long established (77), but the range of hypoxia particularly across the different molecular subtypes needs to be understood. Recording hypoxia within tumors requires obsessive attention to imaging technique, adherence to imaging protocols such as those mandated by the Quantitative Imaging Biomarkers Alliance, and as with all biomarker studies, an establishment and understanding of the reproducibility of the measurement (78, 79). Despite these restrictions, when implemented according to protocol, hypoxia imaging with MRI can be a simple add-on to the staging examination of endometrial cancer. The additional imaging time of 10 mins for a BOLD evaluation and 10 mins for a TOLD evaluation is clinically achievable and can even be implemented in conjunction to increase the robustness of hypoxia evaluation. Methods such as MR fingerprinting may in future also allow simultaneous acquisition of both the T2* and T1 information (80) before, during and after the oxygen challenge, markedly reducing image acquisition time. The derived information is a useful adjunct to that already available and has the potential to substantially alter and enhance the management options offered to patients (**Figure 1**).

## Author contributions

All authors contributed to the design, drafting, writing and editing of this opinion piece.

## Funding

AC, JPBO'C and PJH are supported by the NIHR Manchester Biomedical Research Centre.

## Conflict of interest

The authors declare that the research was conducted in the absence of any commercial or financial relationships that could be construed as a potential conflict of interest.

## Publisher’s note

All claims expressed in this article are solely those of the authors and do not necessarily represent those of their affiliated organizations, or those of the publisher, the editors and the reviewers. Any product that may be evaluated in this article, or claim that may be made by its manufacturer, is not guaranteed or endorsed by the publisher.
